# Clinical outcome of elderly patients (≥ 70 years) with esophageal cancer undergoing definitive or neoadjuvant radio(chemo)therapy: a retrospective single center analysis

**DOI:** 10.1186/s13014-018-1044-8

**Published:** 2018-05-16

**Authors:** Franziska Walter, David Böckle, Nina-Sophie Schmidt-Hegemann, Rebecca Köpple, Sabine Gerum, Stefan Boeck, Martin Angele, Claus Belka, Falk Roeder

**Affiliations:** 1 Department of Radiation Oncology, University Hospital LMU Munich, Marchioninistr 15, 81377 Munich, Germany; 2 Department of Internal Medicine III, University Hospital LMU Munich, Marchioninistr, 15, 81377 Munich, Germany; 3 Department of Surgery, University Hospital LMU Munich, Marchioninistr, 15, 81377 Munich, Germany; 4 Department of Molecular Radiation Oncology, German Cancer Research Center (DKFZ), Heidelberg, Germany

**Keywords:** Elderly, Esophageal cancer, Chemoradiation

## Abstract

**Background:**

To analyse the outcome of elderly patients (≥70 years) with esophageal cancer treated with curative intent radio(chemo)therapy.

**Methods:**

Fifty five patients (median 75 years) receiving curative intent radio(chemo)therapy for esophageal cancer from 1999 to 2015 were retrospectively analyzed. Most patients showed locally advanced disease (T3/4:78%, N+:58%) with squamous cell histology (74%). Charlson comorbidity score was > 1 in 27%. 48 patients (87%) received definitive treatment while 7 patients were treated neoadjuvantly. RT was carried out as 3D-conformal treatment or IMRT. Concurrent chemotherapy was applied in 85%, mainly cisplatin/5-FU or mitomycin/5-FU. ^18^FDG-PET/CT staging was used in 65%.

**Results:**

Median follow-up was 11 months (1–68) and 21 months in survivors. 1- and 2-year rates of LRC, DC, FFTF and OS were 60%/45, 81%/72, 55%/41 and 46%/26% for the entire cohort. In univariate analysis, addition of surgery was associated with improved LRC and FFTF, nodal involvement with improved DC and lower T stage, lower Charlson score and use of PET-CT with improved OS. In multivariate analysis, lower T stage and lower Charlson score remained significant for OS. Patients treated after 2008 showed a significantly improved FFTF (1-year FFTF 64% vs 35%) and OS (1-year OS 66% vs 24%). Maximum (chemo)radiation related grade3+ toxicity was observed in 80% including 7 deaths (13%). Grade5 toxicity was significantly associated with Charlson score (CS > 1:33% vs CS ≤ 1:5%) and treatment period (24% before vs 3% after 2008). The patients treated after 2008 included significantly more SCCs, less T4 stages, had a higher percentage of PET-CT staging and were treated with smaller field lengths. Trends were also observed for lower Charlson scores and increased use of IMRT.

**Conclusion:**

Curative intent (chemo)radiation of elderly patients with esophageal cancer may result in considerable toxicity and unfavorable outcome. However, a clear improvement over time was observed in our cohort, probably based on improved patient selection. In patients with less advanced stages and lower comorbidity similar results as in younger cohorts seem achievable with modern staging and treatment approaches. Age per se should not be a decisive factor, but careful attention should be paid regarding patient selection including a structured and tight follow-up strategy.

## Background

The overall life expectancy in western countries has improved over the last decades while the incidence for cancer has increased. Therefore the management of elderly cancer patients will become a major challenge in the future. For esophageal cancer a rising incidence worldwide has been reported [1] with a considerable change in the morphologic appearance of the tumors. To date, squamous cell carcinomas still account for the majority of esophageal cancers in western countries, however in recent years the incidence of adenocarcinomas has increased notably [2]. Multimodality treatment is considered standard of care in the treatment of patients with locally advanced esophageal cancer based on the encouraging evidence that in potentially curable disease preoperative chemoradiotherapy (CRT) can improve survival of patients with esophageal or esophagogastric-junction (GEJ) cancer [3] compared to surgery alone. In case of non-resectable tumors, definitive CRT may result in 5-year survival rates of approximately 20% [4]. The addition of surgery to CRT has shown to improve local control but not survival in locally advanced esophageal cancer [5]. However, elderly patients are often excluded or at least underrepresented in randomized trials, thus questioning the general transferability of their results into the elderly population [6, 7]. For example, the upper age limit for eligibility in the mentioned CROSS trial was 75 years and the mean age of the included patients was 60 years. While it may be true that some elderly patients are not suitable for multimodality treatment, age per se may not be a good parameter for treatment decisions. However, a recent SEER-analysis on elderly esophageal cancer patients reported a distinct underutilization of treatment in patients aged > 65 years, despite showing a significant survival benefit for patients with treatment vs best supportive care [8]. Further on, increased age did not significantly influence overall outcome or the complication rate in patients treated with extended esophagectomy according to a recent analysis by Pultrum et al. [9]. In contrast, the presence of comorbidity had a significant impact on survival, indicating that comorbidity and/or performance status seem to be more suitable factors for decision making than chronologic age per se. To our knowledge, no high-level evidence specifically addressing the outcome of elderly patients treated by radiation-containing curative intent approaches has been published so far. Here we present a retrospective analysis of our experience including an evaluation of possible factors affecting outcome and toxicity.

## Methods

We performed a retrospective analysis of 55 patients aged ≥70 years with esophageal cancer who were treated with neoadjuvant or definitive RT with or without concomitant chemotherapy at our institution between 11/1999 and 01/2014. Inclusion criteria were newly diagnosed histologically proven squamous cell or adenocarcinoma of the esophagus, age ≥ 70 years at start of therapy and neoadjuvant or definitive RT using 3D-conformal or intensity-modulated RT techniques. Patients were excluded if they had distant metastases other than celiac or supraclavicular lymph nodes, were treated for recurrent disease or received 2-dimensional radiation therapy. Median age was 75 years (range 70–85), 45 patients were male, 10 were female. The majority of patients received definitive RT or CRT, 7 patients received surgery after neoadjuvant CRT. A systematic review of patients’ clinical charts and reports was performed to obtain patient and treatment characteristics, reported toxicity, and treatment outcome. For the purpose of this study, all patients were restaged according to the 7th edition of the UICC TNM classification. For detailed patient and treatment characteristics see Table 1.

**Table 1:** Patient and treatment characteristics (n)

Gender		Time period	
Male	45	Before 2008	25
Female	10	After 2008	30
Age		PET-CT Staging	
Median	75 yrs	Yes	36
Range	70–85 yrs	No	19
Charlson Comorbidity Index		RT sequence	
≤1	40	Definitive	48
> 1	15	Neoadjuvant	7
Histology		RT technique	
Adenocarcinoma	14	3D-CRT	50
SCC	41	IMRT	5
Localization		RT dose	
Cervical	5	median	59.4Gy
Upper thoracic	12	range	10.8-66Gy
Middle thoracic	20		
Lower thoracic	18	Chemotherapy	
		Yes	47
Grading		full course	31
G1	1	> 80%	36
G2	30	< 80%	11
G3	24	None	8
T stage		N stage	
T1	1	N0	23
T2	11	N+	32
T3	32		
T4	11		

*yrs* years, *SCC* squamous cell carcinoma, *PET-CT* positron emission tomography with computed tomography, *RT* radiation therapy, *3D-CRT* three-dimensional conformal radiation therapy, *IMRT* intensity modulated radiation therapy

### Initial work-up

Initial work-up included at least clinical and laboratory examinations, endoscopy with biopsy and computed tomography of the chest and abdominal ultrasound. 36 patients received ^18^FDG positron emission tomography (PET/CT) imaging prior to radiation therapy.

### (chemo)-radiation therapy

Due to the long time period covered by this study, target delineation, radiation technique and dose prescription varied to some extent. Usually patients were treated in supine position using an alpha-cradle. The gross tumor volume (GTV) included the primary tumor and involved lymph nodes. In general, the primary CTV covered at least 5 cm in both directions along the longitudinal axis from the primary tumor and at least 1 cm of uninvolved mediastinal soft tissue in axial direction excluding adjacent lung tissue in the sense of an elective nodal irradiation. An isotropic margin of 0.5–1 cm was added to receive the PTV. Primary tumor and lymph node delineation was further supported by PET-CT if available (*n* = 36). Radiation therapy was administered in multiple field techniques either as 3D-conformal RT (*n* = 50, 91%) or IMRT (*n* = 5, 9%) with an intended dose of 50.4 Gy in conventional fractionation. For definitive treatment a sequential boost of 9 Gy to the GTV plus 2 cm in longitudinal direction and 1 cm in axial direction was usually added. Patient treated with IMRT received a similar dose prescription using an integrated boost concept. Patients were scheduled for simultaneous chemotherapy (*n* = 47, 85%) or radiation alone (*n* = 8, 15%) based on performance status, comorbidity and presence of specific contraindications regarding the planned substances by the treating radiation oncologist. In the majority of patients, chemotherapy consisted either of two courses of cisplatin/5-fluorouracil (cisplatin 75 mg/m^2^ body-surface area on the first day combined with 5-fluorouracil 1000 mg/m^2^ continuous infusion daily for four days) or two courses of mitomycin C/5-fluorouracil (mitomycin C 10 mg/m^2^ day one, 5-fluorouracil 1000 mg/m^2^ first 5 days).

### Follow-up

Follow-up took place at our institution or at the referring center and included at least clinical examination and endoscopy. In case of clinical evidence for loco-regional recurrence or distant spread, additional tests or imaging modalities were performed to confirm or exclude disease progression at the discretion of the treating physician. Since 2008, all patients were offered a structured follow-up at our institution including at least clinical examination, endoscopy and CT chest every three months for the first two years and in 6–12 month intervals thereafter. Patients and their primary care physicians (via treatment report) were encouraged to report complications after chemoradiation promptly to our center and were contacted via phone in case of missing a scheduled follow-up visit.

### Statistical and ethical considerations

All time to event data was calculated from the date of first radiation treatment until last follow-up information or until death using the Kaplan-Meier method. Locoregional control (LRC) was defined as absence of disease progression in the primary tumor region or regional lymph nodes. Distant control (DC) was defined as absence of distant failure. Freedom from treatment failure (FFTF) was defined as absence of locoregional and distant failure. Differences between subgroups regarding time to event data were compared using a log rank test for univariate analysis. Parameters with *p* < 0.1 in univariate analysis were entered into a Cox regression model for multivariate analysis. Differences in patient- or treatment related parameters between subgroups defined by treatment period were analyzed using the chi-square or fisher’s exact test for categorial and the t-test for continuous variables. A *p*-value of < 0.05 was defined as statistically significant. Acute and late toxicity was scored retrospectively according to CTCAEV4.03. Comorbidity was scored according to the Charlson score. Histopathological regression in resected patients was graded according to Baldus et al. [10]. The study was performed in accordance to the declaration of Helsinki in its latest version and was approved by our independent ethics committee. All patients gave written informed consent prior to treatment initiation.

## Results

Median follow up for the entire cohort was 11 months (1–68) and 21 months in survivors. Radiotherapy was completed as planned without interruptions ≥4 days in 47 patients (85%). In patients scheduled for combined CRT, 66% received full course chemotherapy and 77% received at least 80% of the planned chemotherapy dose.

### Surgery and pathologic response

Seven patients were treated neoadjuvantly and received surgery. Of these, one suffered from adenocarcinoma and six from squamous cell carcinoma. Margin status was available in 5 patients and was microscopically complete in all of them. Response status according to imaging was complete remission in one, partial remission in 4 and stable disease in 1. Histopathological regression grading was available in 5 patients and resulted in grade 2 in 2 patients, grade 3 in 1 patient and grade 4 (pathologically complete remission) in 2 patients.

### Outcome

**Fig. 1:**
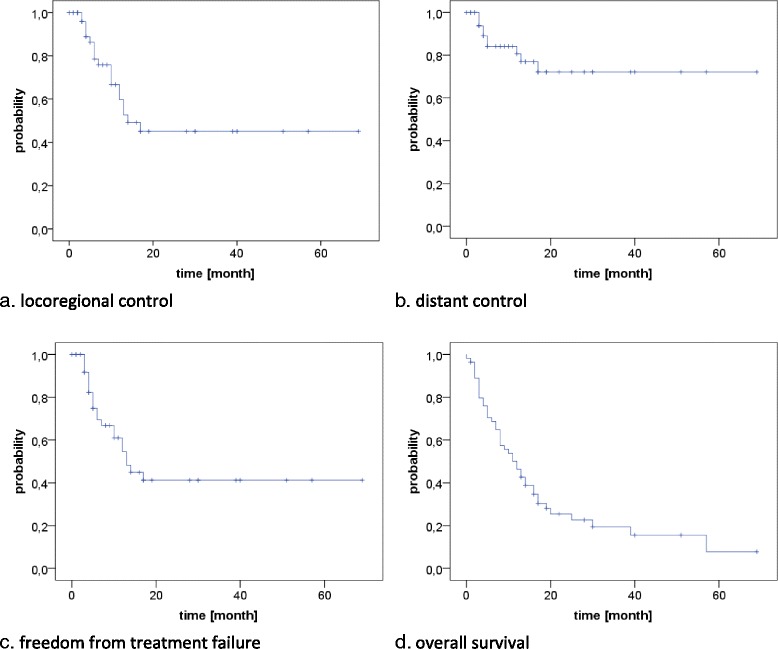
LRC, DC, FFTF, OS for the entire cohort

**Fig. 2:**
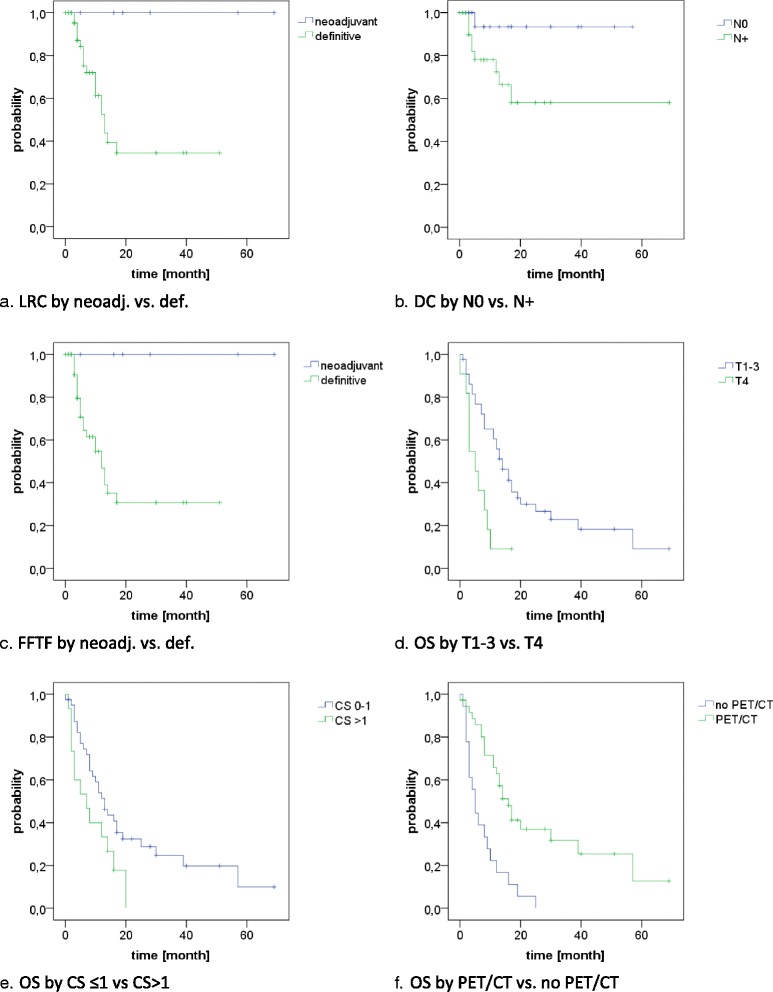
prognostic factors for LRC, DC, FFTF and OS according to univariate analysis

**Fig. 3:**
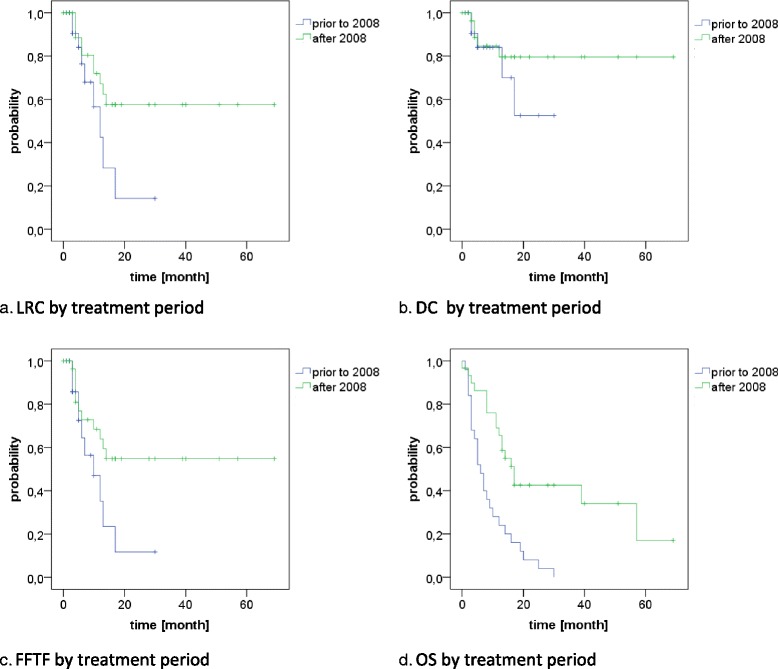
LRC, DC, FFTF, OS according to treatment period (before vs after 2008)

Locoregional recurrences were observed in 19 patients, translating into estimated 1- and 2-year LRC rates of 60 and 45% (Fig. 1). Of these 14 were only local, two were only nodal and three were combined. The median time to the onset of a locoregional recurrence was 7 months. In univariate analysis only the addition of surgery resulted in significantly improved locoregional control (1-year LRC 100% vs. 53%, 2-year LRC 100% vs. 34%, *p* = 0.02, Fig. 2). A trend for improved locoregional control was found in patients treated in the recent time period (after 2008) (1-year LRC 67% vs. 42%, 2-year LRC 58% vs. 14%, *p* = 0.06, Fig. 3) and for patients staged with PET-CT (1-year LRC 66% vs. 27%, 2-year LRC 54% vs. 0%, *p* = 0.085), see Table 2.

**Table 2:** Univariate analysis of prognostic factors

	LRC		DC		FFTF		OS	
	1-yr rate	*p* value	1-yr rate	*p* value	1-yr rate	*p* value	1-yr rate	*p* value
Gender								
Male	63%	0.192	81%	0.464	57%	0.33	43%	0.443
Female	44%		78%		44%		60%	
Age								
< 75 yrs	54%	0.523	78%	0.572	50%	0.652	30%	0.236
≥ 75 yrs	63%		80%		57%		58%	
Charlson score								
≤ 1	61%	0.901	83	0.493	58%	0.991	51%	**0.041**
> 1	56%		74		55%		33%	
Histology								
SCC	62%	0.484	86%	0.121	57%	0.456	50%	0.107
Adenocarcinoma	55%		63%		47%		36%	
Localization								
Cervical	80%	0.335	80%	0.427	60%	0.267	60%	0.249
Upper thoracic	71%		100%		71%		33%	
Middle thoracic	51%		76%		47%		47%	
Lower thoracic	59%		77%		53%		61%	
Grading								
G2	63%	0.853	80%	0.811	57%	0.818	53%	0.238
G3	53%		79%		48%		39%	
T stage								
T1–3	59%	0.911	83%	0.374	55%	0.559	56%	**0.002**
T4	78%		78%		67%		9%	
N stage								
N0	70%	0.201	93%	**0.035**	64%	0.161	44%	0.921
N+	54%		72%		49%		48%	
time period								
before 2008	42%	0.061	84%	0.366	35%	**0.048**	24%	**< 0.001**
after 2008	67%		80%		64%		66%	
PET-CT Staging								
yes	66%	0.085	80%	0.866	61%	0.101	63%	**< 0.001**
no	27%		86%		25%		17%	
RT sequence								
neoadj	100%	**0.022**	100%	0.146	100%	**0.014**	71%	0.055
definitive	53%		77%		47%		43%	
RT technique								
3D-CRT	59%	0.638	83%	0.918	53%	0.508	43%	0.076
IMRT	75%		75%		75%		80%	
Chemotherapy								
no	30%	0.351	86%	0.481	28%	0.24	50%	0.317
yes	64%		80%		61%		46%	
LRC								
no			87%	0.054			37%	0.605
yes			72%				63%	

*LRC* locoregional control, *DC* distant control, *FFTF* freedom from treatment failure, *OS* overall survival, *1-yr rate* 1-year rate, *yrs.* years, *PET-CT* positron emission tomography with computed tomography, *RT* radiation therapy, *3D-CRT* three-dimensional conformal radiation therapy, *IMRT* intensity-modulated radiation therapy

Distant metastases were observed in 10 patients, mainly to the lung (*n* = 7), translating into estimated 1- and 2-year DC rates of 81 and 72% (Fig. 1). The median time to the onset of distant failure was 8 months. In univariate analysis, nodal involvement was the only significant predictor of distant failure (1-year DC 93% vs. 72%, 2-year DC 93% vs. 58%, *p* = 0.04, Fig. 2), while a trend to improved DC was observed in patients with the achievement of locoregional control (1-year DC 87% vs. 72%, 2-year DC 87% vs. 51%, *p* = 0.054), see Table 2.

Disease progression in general was found in 22 patients, translating into estimated 1- and 2-year FFTF rates of 55 and 41% (Fig. 1). Of these, 12 were isolated locoregional, 3 were isolated distant and 7 were combined. In univariate analysis, the addition of surgery (1-year FFTF 47% vs. 100% and 2-year FFTF 31% vs. 100%, *p* = 0.02, Fig. 2) and treatment in the latter time period (1-year FFTF 35% vs. 64%, 2-year FFTF 12% vs 55%, *p* = 0.048, Fig. 3) resulted in significantly improved FFTF (Table 2).

At time of analysis, 43 patients had died (78%), translating into a median overall survival of 12 months with estimated 1- and 2-year OS rates of 46 and 26% (Fig. 1). In univariate analysis, OS was significantly associated with T stage (1-year OS T1–3: 56% vs T4: 9%, *p* = 0.002, Fig. 2), comorbidity (1-year OS CS ≤1: 51% vs. CS > 1: 33%, p = 0.04, Fig. 2), treatment period (1-year OS prior 2008: 24% vs after 2008: 66%, *p* < 0.001, Fig. 3) and the use of PET-CT for staging (1-year OS 63% vs 17%, *p* < 0.001, Fig. 2). A trend was found for the addition of surgery (1-year OS 71% vs 43%, 2-year OS 57% vs 20%, *p* = 0.055) and the use of IMRT (1-year OS 80% vs 43%, *p* = 0.076), see Table 2.

In multivariate analysis none of the tested factors was significant for LRC, DC or FFTF. However for overall survival T stage (*p* = 0.029, HR 2.97, KI 1.12–7.92) and comorbidity (*p* = 0.007, HR 2.93, KI 1.33–6.42) remained significant in the multivariate analysis.

### Toxicity

Maximum acute toxicity during or after (chemo)radiation therapy was grade 3 in 31 patients (56%), grade 4 in 6 (11%) and grade 5 in 7 (13%), mainly dysphagia, infections and leukopenia. For detailed analysis of acute toxicity see Table 3. Three patients died during hospital admission for CRT (2 sepsis, 1 hemorrhage) and four patients died after CRT was finished and they had been discharged (3 pneumonia, 1 sepsis). None of these four patients had been readmitted to our center for complication management. Grade 5 toxicity correlated significantly with treatment period (24% before 2008 vs. 3% after 2008, *p* = 0.039) and comorbidity (33% with Charlson score > 1 vs. 5% with Charlson score ≤ 1, *p* = 0.013). Of the 7 patients who received additional surgery, two developed grade 4 postoperative complications (sepsis). 30-day mortality was 14% (1 patient died intraoperatively due to cardiac failure).

**Table 3:** acute toxicities

	All grades		Grade 3–5	
	n	%	n	%
non-hematological				
skin	16	29	0	0
mucositis	23	42	2	4
dysphagia	52	95	32	58
nausea	11	20	1	2
vomiting	4	7	1	2
diarrhea	5	9	0	0
weight loss	10	18	0	0
hoarsness	10	18	0	0
bleeding	1	2	1	2
others	7	9	5	9
hematological				
anemia	50	91	3	5
leucopenia	46	84	18	33
thrombopenia	36	65	4	7
infection	20	36	16	29
renal insufficiency	13	24	0	0

Late toxicity in terms of dysphagia with the need for intervention was observed in 13 patients. Of those one was long-term PEG dependent, 12 needed dilatations (median 2, range 1–6) and one stent placement. No cases of severe pulmonary or cardiac late toxicity were observed.

### Treatment period

We analyzed our cohort with regard to different treatment periods to evaluate if outcome has improved over time. Because of the implementation of a structured follow-up in 2008, we splitted the cohort in subgroups treated before and after 2008. Patients treated in the recent time period (since 2008) showed improved outcome with regard to 1-year LRC (42% vs. 67%), DC (80% vs. 84%y), FFTF (35% vs. 64%y) and OS (24% vs. 66%y), with the difference in FFTF and OS reaching statistical significance (Fig. 3, Table 2). We therefore evaluated possible differences regarding patient and treatment related factors between those groups. They differed significantly with regard to histology (56% vs 90% SCC, *p* = 0.04), T stage 4 (32% vs 10%, *p* = 0.042), number of patients receiving PET-CT for treatment planning (25% vs 100%, *p* < 0.001) and maximum cranio-caudal extension of the PTV (mean 21.6 cm vs 19.0 cm, *p* = 0.017). Trends were found for Charlson score ≤ 1 (60% vs 83, *p* = 0.053) and the use of IMRT (0% vs. 17%, *p* = 0.056), see Table 4. As mentioned before, also grade 5 toxicity correlated significantly with treatment period (24% vs. 3%, *p* = 0.039).

**Table 4:** Treatment period

	Before 2008		After 2008		
	n	%	n	%	*p*-value
Gender					
male	22	88	23	77	0.318
female	3	12	7	23	
Age					
< median (75 yrs)	8	32	15	50	0.178
≥ median	17	68	15	50	
Charlson score					
≤ 1	15	60	25	83	0.053
> 1	10	40	5	17	
Histology					
SCC	14	56	27	90	**0.04**
Adenocarcinoma	11	44	3	10	
Localization					
Cervical	2	8	3	10	0.964
Upper thoracic	5	20	7	23	
Middle throacic	9	36	11	37	
Lower thoracic	9	36	9	30	
Grading					
G2	13	52	17	57	0.580
G3	12	48	12	40	
T stage					
T1–3	17	68	27	90	**0.042**
T4	8	32	3	10	
N stage					
N0	11	44	12	40	0.765
N+	14	56	18	60	
PET-CT Staging					
yes	6	24	30	100	**< 0.001**
no	19	76	0	0	
RT sequence					
Neoadjuvant	1	4	6	20	0.112
Definitive	24	96	24	80	
RT technique					
3D-CRT	25	100	25	83	0.056
IMRT	0	0	5	17	
Chemotherapy					
yes	19	76	28	93	0.123
no	6	24	2	7	
Treatment field					
< 20 cm	7	28	18	60	**0.033**
≥ 20 cm	16	64	12	40	

*yrs* years, *SCC* squamous cell carcinoma, *PET-CT* positron emission tomography with computed tomography, *RT* radiation therapy, *3D-CRT* three-dimensional conformal radiation therapy, *IMRT* intensity-modulated radiation therapy, *cm* centimeters

## Discussion

The treatment of locally advanced non-metastasized esophageal cancer usually consists of surgery, chemoradiation or combinations of both [11]. Efficacy and tolerability of definitive concurrent chemoradiation and its benefits in terms of survival compared to radiation alone have been established by randomized trials already in the 1990s [12]. More recently preoperative CRT has been shown to improve survival of patients with potentially curable esophageal or GEJ-cancer [3] compared to surgery alone. However, elderly patients are generally underrepresented or even excluded from such trials [6, 13] resulting in less high-level evidence regarding their appropriate treatment. Combined with other factors (like the (mis)-conception of decreased treatment tolerance per se, multiple comorbidities or socioeconomic problems), this uncertainty may result in considerable undertreatment with reduced survival as recently shown by Molena et al. [8].

In our study we analyzed 55 patients treated mainly with definitive or (to a lesser extent) neoadjuvant (chemo)radiation, which are both widely accepted as curative intent treatment options. With this approach we observed a median survival of 12 months with 2-year LC and OS rates of 45 and 26%. These results compare less favorable with large randomized studies including younger populations, which report a median survival of 12–19 months with 2-year LRC rates of 41–57% and 2-year-OS rates of 28–40% using similar definitive chemoradiation schemes [5, 12, 14, 15]. The SCOPE1 trial recently reported even much more favourable outcomes in their standard arm using definitive chemoradiation leading to a median OS of 25 months with a 2-year OS rate of 56% [16]. In contrast, a population-based study from the Netherlands including patients from four referral centers treated by radio(chemo)therapy observed very similar results with a median survival of 11 months and 2-year LRC and OS rates of 45 and 22%, respectively [17], indicating that results generated in the general population may vary distinctly from outcomes in controlled trials. Several other groups have specifically evaluated the outcome of elderly patients with esophageal cancer treated with chemoradiation. They observed median OS times of 13–19 months and 2-year OS rates of 27–43% in more or less selected patient cohorts [18–24]. Servagi-Vernat et al. [24] performed the only prospective phase II trial and found a 3-year OS of 22% in patients aged ≥75 years treated with chemoradiation consisting of 50 Gy and single-agent cisplatin or oxaliplatin, respectively. Vlacich et al. [25] reported a national cancer database (NCDB) analysis focusing on treatment utilization and outcome in patients aged ≥70 years and reported a 2-year OS rate of roughly 35% for patients treated by chemoradiation. Regarding the influence of age per se, conflicting results have been described in series directly comparing elderly patients with younger ones. For example, Vonken et al. [26] compared 76 patients aged ≥70 yrs. with 176 patients aged < 70 years treated either by neoadjuvant or definitive chemoradiation and found no significant difference in overall survival. In contrast, Takeuchi et al. [20] reported on 33 patients aged > 71 years and 145 patients aged < 70 years treated with definitive chemoradiation and observed a clearly inferior median survival in the elderly group (median OS 14.7 vs 35.1 months). Interestingly, both series reported lower treatment compliance (more chemotherapy dose reductions and/or discontinuations) and Takeuchi et al. [20] found an increased toxicity in the elderly group. We also observed considerable toxicity in our series including treatment-related deaths in 7 patients, which mainly occurred after patients had been discharged from hospital after treatment was finished. Overall survival and grade 5 toxicities were significantly associated with a Charlson score > 1, indicating that comorbidity may play a more important role than age per se. Tougeron et al. [22] similarly described a significant association of comorbidity with treatment tolerance and overall survival in their series of 109 patients aged ≥70 treated with definitive chemoradiation. In the prior mentioned NCDB analysis, only 5% of the patients had a Charlson score > 1 compared to 27% in our study, which may have contributed to the favourable results [25]. Similarly, in the SCOPE1 trial only 15% of the patients included received definitive chemoradiation due to comorbidities and all had good performance status [16]. Regarding specific comorbidities we did not observe a significant correlation to outcome or grade 5 toxicity, although patients with grade 5 toxicity showed more often cardiovascular comorbidities (57% vs 38%, data not shown). Interestingly, we found large differences in overall outcome and toxicity if patients were stratified according to treatment period. Patient who received treatment after 2008 had a clearly improved outcome with a 1-year LRC and OS rates of 67 and 66% compared to 42 and 24% if treatment started before 2008. Moreover, grade 5 toxicity dropped from 24 to 3%. Comparing the subgroups according to treatment period we observed significantly less advanced T stages (T4 10% vs. 32%) and adenocarcinomas (10% vs. 44%), a significantly increased percentage of patients staged with PET-CT (100% vs. 25%) and a significantly reduced craniocaudal extension of the PTV in recently treated patients. Trends were also observed for the increasing use of IMRT and a lower Charlson score. High T stage is well known to be associated with decreased survival as shown by others [18, 27, 28] and was also identified as a negative prognostic for OS in our series according to multivariate analysis. Regarding histology, a clear impact on overall survival as not been established so far. While a recent population-based analysis [17] found a significantly decreased 2-year survival in patients with adenocarcinoma compared to SCC (17% vs 29%) after definitive CRT or RT, a large retrospective cohort analysis for patient treated with chemoradiation could only confirm an increased risk for distant metastases but did not observe a difference in overall survival [29]. Charlson score has also been shown to be associated with overall survival as well as with grade 5 toxicity in our series. Similarly, Tougeron et al. [22] observed a significant impact of Charlson score on treatment tolerance, high grade adverse effects and overall survival in their series of elderly patient treated with chemoradiation. Taken together, it seems that a better selection of patients has contributed to the clearly improved outcome in the latter time period. This selection process may have been supported by the increased utilization of PET-CT for staging prior to treatment initiation (100% vs. 25%), which was associated with improved survival in our series according to univariate analysis although not confirmed in multivariate analysis. Similarly, Metzger et al. [30] recently described an association of PET-CT use with improved local-recurrence free survival and overall survival in their series of 145 patients treated with definitive or neoadjuvant chemoradiation. Treatment-related parameters like modern radiation techniques (IMRT), smaller field length and a more structured follow-up strategy may also have contributed to improved outcome and reduced high grade toxicity in the recently treated patients, although we could not confirm a significant impact of one of those factors independently in our analysis. However, several groups have shown not only dosimetric advantages but also reduced toxicity and sometimes even improved locoregional control and survival with IMRT compared to 3D—conformal approaches [31–33]. A reduction of field length in terms of “involved field irradiation (IFI)” instead of “elective nodal irradiation (ENI)” has been a matter a debate for nearly a decade because of its suggested reduction in toxicity. A recent meta-analysis confirmed a significant reduction of toxicity and observed no significant differences in local control or survival with reduced target volumes [34]. Moreover, Jing et al. [35] observed a reduction in toxicity but no difference in OS comparing IFI with ENI in a cohort of patients aged ≥70 years and concluded that IFI should be preferred especially in elderly patients. Taken together, our clearly superior results in the latter time period seemed to be based on a combination of improved patient selection and an adaption of treatment towards less toxic approaches. Interestingly, an indirect comparison of more recent and older prospective trials in unselected patients leads to a similar direction. For example the standard arm of the SCOPE1 trial (started in 2008) reported much better median OS (25 months) and 2-year OS (56%) compared to the standard arm of RTOG 8501 (recruiting patients in the late 80s) with a median OS 12.5 months and a 2-year OS of 38%, although the chemoradiation regimen used in both arms was similar at least regarding radiation dose and chemotherapy drugs [12, 16]. We also observed a nearby doubled median overall survival comparing our patients from the recent and the previous time period indicating that probably a combination of several advances including patient selection, radiation technique, field design and supportive care may have contributed to the improvement over time. With respect to those boundaries, similar results can be achieved in elderly patients regarding toxicity and outcome compared to unselected cohorts as shown by the results in our recently treated patient group.

Of course our study has some limitations, namely its retrospective nature, small sample size, short follow-up and inhomogeneous patient and treatment characteristics. However, in the absence of randomized trials specifically addressing the value of chemoradiation in elderly patient cohorts we feel that it adds valuable information to the existing body of literature.

## Conclusion

In summary, curative intent treatment of elderly patients with definitive or neoadjuvant chemoradiation may result in considerable toxicity and less favorable outcome. Despite the disappointing results regarding the entire cohort, we observed a clear improvement with increased survival and reduced toxicity over time, mainly based on patient selection and treatment adaptions. In patients with less advanced disease stage and lower comorbidity, which are staged and treated with modern techniques and concepts, similar results compared to younger cohorts seem to be achievable. Therefore age per se should not be a decisive factor in treatment decisions in esophageal cancer although careful attention has to be paid regarding patient selection for potentially curative treatments including a structured and tight follow-up strategy.

## References

[1] Enzinger PC, Mayer RJ (2003). Esophageal cancer. N Engl J Med.

[2] Arnold M, Soerjomataram I, Ferlay J, Forman D (2015). Global incidence of oesophageal cancer by histological subtype in 2012. Gut.

[3] Hagen V, Hulshof MC, van Lanschot JJ (2012). Preoperative chemoradiotherapy for esophageal or junctional cancer. N Engl J Med.

[4] Stahl M, Budach W (2017). Definitive chemoradiotherapy. J Thorac Dis.

[5] Stahl M, Stuschke M, Lehmann N (2005). Chemoradiation with and without surgery in patients with locally advanced squamous cell carcinoma of the esophagus. J Clin Oncol.

[6] Hutchins LF, Unger JM, Crowley JJ (1999). Underrepresentation of patients 65 years of age or older in cancer-treatment trials. N Engl J Med.

[7] Andrä C, Klein A, Dürr HR (2017). External-beam radiation therapy combined with limb-sparing surgery in elderly patients (>70 years) with primary soft tissue sarcomas of the extremities: a retrospective analysis. Strahlenther Onkol.

[8] Molena D, Stem M, Blackford AL, Lidor AO (2017). Esophageal cancer treatment is underutilized among elderly patients in the USA. J Gastrointest Surg.

[9] Pultrum BB, Bosch DJ, Nijsten MW (2010). Extended esophagectomy in elderly patients with esophageal cancer: minor effect of age alone in determining the postoperative course and survival. Ann Surg Oncol.

[10] Baldus SE, Mönig SP, Schröder W (2004). Regression of oesophageal carcinomas after neoadjuvant radiochemotherapy: criteria of the histopathological evaluation. Pathologe.

[11] Hulshof MC, van Laarhoven HW (2016). Chemoradiotherapy in tumours of the oesophagus and gastro-oesophageal junction. Best Prac Res Clin Gastroenterol.

[12] Herskovic A, Martz K, al-Sarraf M (1992). Combined chemotherapy and radiotherapy compared with radiotherapy alone in patients with cancer of the esophagus. N Engl J Med.

[13] Scher KS, Hurria A (2012). Under-representation of older adults in cancer registration trials: known problem, little progress. J Clin Oncol.

[14] Minsky BD, Pajak TF, Ginsberg RK (2002). INT 0123 (radiation therapy oncology group 94-05) phase III trial of combined-modality therapy for esophageal cancer: high-dose versus standard-dose radiation therapy. J Clin Oncol.

[15] Bedenne L, Michel P, Bouche O (2007). Chemoradiation followed by surgery compared with chemoradiation alone in squamous cancer of the esophagus: FFCD 9102. J Clin Oncol.

[16] Crosby T, Hurt CN, Falk S (2013). Chemoradiotherapy with or without cetuximab in patients with oesophageal cancer (SCOPE1): a multicenter, phase 2/3 randomised trial. Lancet Oncol.

[17] Smit JK, Mujis CT, Burgerhof JG (2013). Survival after definitive (chemo)radiotherapy in esophageal cancer patients: a population-based study in the north-East Netherlands. Ann Surg Oncol.

[18] Zhao L, Zhou Y, Pan H (2017). Radiotherapy alone or concurrent chemoradiation for esophageal squamous cell carcinoma in elderly patients. J Cancer.

[19] Münch S, Heinrich C, Habermehl D (2017). Primary radio(chemo)therapy for esophageal cacer in elderly patients: are efficiency and toxicity comparable with younger patients?. Eur J Med Res.

[20] Takeuchi S, Ohtsu A, Doi T (2007). A retrospective study of definitive chemoradiotherapy for elderly patients with esophageal cancer. Am J Clin Oncol.

[21] Li X, Zhao LJ, Liu NB (2015). Feasibility and efficacy of concurrent chemoradiotherapy in elderly patients with esophageal squamous cell carcinoma: a retrospective study of 116 cases from a single institution. Asian Pac J Cancer Prev.

[22] Tougeron D, di Fiore F, Thureau S (2008). Safety and outcome of definitive chemoradiotherapy in elderly patients with oesophageal cancer. Br J Cancer.

[23] Qu X, Biagi J, Banashkevich A (2015). Management and outcomes of localized esophageal and gastroesophageal junction cancer in older patients. Curr Oncol.

[24] Servagi-Vernat S, Crehange G, Roullet et al. Phase II study of a platinum-based adapted chemotherapy regimen combined with radiotherapy in patients 75 years and older with esophageal cancer. Drugs Aging 2015;32:487–493. 10.1007/s40266-015-0275-8 26038198

[25] Vlacich G, Samson PP, Perkins SM (2017). Treatment utilization and outcomes in elderly patients with locally advanced esophageal carcinoma: a review of the National Cancer Database. Cancer Medicine.

[26] Voncken FE, van der Kaaij RT, Sikorska K, et al. Advanced age is not a contraindication for treatment with curative intent in esophageal cancer. Am J Clin Oncol. 2017; epub ahead of print 10.1097/COC.0000000000000390 28763327

[27] Häfner MF, Lang K, Krug D (2015). Prognostic factors, patterns of recurrence and toxicity for patients with esophageal cancer undergoing definitive radiotherapy or chemo-radiotherapy. J Radiat Res.

[28] Nomura M, Shitara K, Kodaira T (2012). Recursive partitioning analysis for new classification of patients with esophageal cancer treated by chemoradiotherapy. Int J Radiat Oncol Biol Phys.

[29] Xi M, Xu C, Liao Z (2017). The impact of histology on recurrence patterns in esophageal cancer treated with definitive chemoradiotherapy. Radiother Oncol.

[30] Metzger JC, Wollschläger D, Miederer M (2017). Inclusion of PET-CT into planning of primary or neoadjuvant chemoradiotherapy of esophageal cancer improves prognosis. Strahlenther Onkol.

[31] Roeder F, Nicolay NH, Nguyen T (2014). Intensity modulated radiotherapy (IMRT) with concurrent chemotherapy as definitive treatment of locally advanced esophageal cancer. Radiat Oncol.

[32] Freilich J, Hoffe SE, Almhanna K (2015). Comparative outcomes for three-dimensional conformal versus intensity-modulated radiation therapy for esophageal cancer. Dis Esophagus.

[33] Lin SH, Wang L, Myles B (2012). Propensity score-based comparison of long-term outcomes with 3-dimenionals conformal radiotherapy vs intensity-modulated radiotherapy for esophageal cancer. Int J Radiat Oncol Biol Phys.

[34] Wang X, Miao C, Chen Z, et al. Can involved-field irradiation replace elective nodal irradiation in chemoradiotherapy for esophageal cancer ? A systematic review and meta-analysis. Onco Targets Ther. 2017; epub ahead of print 10.2147/OTT.S130285 PMC5396978 28442917

[35] Jing W, Zhu H, Guo H (2015). Feasibility of elective nodal irradiation (ENI) ad involved field irradiation (IFI) in radiotherapy for the elderly patients (aged ≥ 70 years) with esophageal squamous cell cancer: a retrospective analysis from a single institute. PLoS One.

